# Association of *UCP1* and *UCP2* variants with diabetic retinopathy susceptibility in type-2 diabetes mellitus patients: a meta-analysis

**DOI:** 10.1186/s12886-021-01838-1

**Published:** 2021-02-12

**Authors:** Xujia Liu, Zehua Jiang, Guihua Zhang, Tsz Kin Ng, Zhenggen Wu

**Affiliations:** 1 Joint Shantou International Eye Center of Shantou University and the Chinese University of Hong Kong, North Dongxia Road, Shantou, 515041 Guangdong China; 2 Shantou University Medical College, Shantou, Guangdong China; 3 Department of Ophthalmology and Visual Sciences, the Chinese University of Hong Kong, Hong Kong Special Administrative Region, China

**Keywords:** Diabetic retinopathy, Uncoupling proteins, Variants, Meta-analysis

## Abstract

**Background:**

Genetic association of uncoupling proteins (UCPs) variants with the susceptibility of diabetic retinopathy (DR) in diabetes mellitus (DM) patients has been reported but with controversy. Here we aimed to conduct a meta-analysis to confirm the association of different UCPs variants with DR.

**Methods:**

Three databases (Medline Ovid, Embase Ovid and CENTRAL) were applied in the literature search. Five genetic models, including allelic, homozygous, heterozygous, dominant and recessive models, were evaluated. Odds ratios (OR) were estimated under the random or fixed-effects models. Subgroup analyses, publication bias and sensitivity analyses were also conducted.

**Results:**

Eleven studies on 2 UCPs variants (*UCP1* rs1800592 and *UCP2* rs659366) were included. Our meta-analysis showed that *UCP1* rs1800592 was not associated with DR in type-2 DM patients, and *UCP2* rs659366 also showed no association with DR. In the subgroup analyses on the stage of DR, allele G of *UCP1* rs1800592 significantly increased the susceptibility of proliferative diabetic retinopathy (PDR) in type-2 DM patients in the allelic (OR = 1.26, *P* = 0.03) and homozygous models (OR = 1.60, *P* = 0.04). Subgroup analysis on ethnicity did not found any significant association of rs1800592 and rs659366 with DR.

**Conclusion:**

Our meta-analysis confirmed the association of *UCP1* rs1800592 variant with PDR in patients with type-2 DM, suggesting its potential as a genetic marker for PDR prediction in population screening.

**Supplementary Information:**

The online version contains supplementary material available at 10.1186/s12886-021-01838-1.

## Background

Diabetic retinopathy (DR), a common sight-threatening microvascular complication among patients with diabetes mellitus (DM), is the major cause of irreversible blindness and visual impairment in working-age adults [1]. Though the pathophysiological mechanisms of DR remain elusive, increasing evidence suggests that long duration of DM, poor control of blood glucose and high blood pressure mainly contribute to the pathogenesis and development of DR [2]. However, DR could also occur in patients with short duration of DM, well control of blood glucose and normal blood pressure. Besides, epidemiological studies revealed familial inheritance and ethnic variations in DR [3], indicating that genetic factors could play a role in the pathogenesis and development of DR [4].

The elevation of oxidative stress has been suggested contributing to the development of DM complications [5], which is caused by reactive oxygen species (ROS) overproduction, mainly the mitochondrial ROS [5–7]. Excessive ROS resulted from hyperglycemia causes retinal mitochondrial dysfunction with serious damage to the oxidative phosphorylation complexes and abolished adenosine triphosphate biosynthesis [8], and induces capillary endothelial cell apoptosis, which subsequently leads to the dysregulation of the angiogenesis-related genes [9] and diabetic microvascular complications, including DR [10, 11]. Ion channels physiologically play a role in signal transmission and visual processing, but also link to induced oxidative stress and significantly contribute to a wide spectrum of ocular diseases [12]. Uncoupling proteins (UCPs) belong to a group of proton carrier transporters (H^+^) in the inner membrane of mitochondria [13]. UCPs are able to uncouple the oxidized substrates and dissipate the potential energy on the inner membrane as heat to reduce ROS overproduction from mitochondria [14–17]. The overproduced ROS could cause increases proton conductance by UCP1–3, leading to decease in superoxide radicals through the mitochondria respiratory chain reaction [18]. In human genome, there were five different UCPs, named UCP1 to 5, with various tissue distributions and functions [19]. Uncoupling protein 1 (*UCP1*) gene is located on chromosome 4q31.1 and found to be expressed in brown adipose tissue, endothelial cells and pericytes of retina [20]. UCP1 mainly plays a role in the maintenance of body temperature in a cold environment through non-shivering thermogenesis [17]. It has been shown that elevated of glucose levels upregulates UCP1 expression, protecting cells from glucose-induced ROS damage [21]. Uncoupling protein 2 (*UCP2*) and 3 (*UCP3*) genes are both located in the same cluster on chromosome 11q13.4. UCP2 is ubiquitously expressed across different tissues in the body, whereas UCP3 is mainly expressed in the skeletal muscle tissue [22]. In *UCP2* knockout mice, ROS production increases in macrophages and pancreatic islets [23, 24], whereas overexpression of UCP2 inhibits mitochondrial death pathway in cardiomyocytes [25], indicating that UCP2 could be involved in cell protection from ROS damage. UCP2 and UCP3, together with *SLC25A27* (UCP4) and *BMCP1* (UCP5), exert cytoprotective effects by reducing oxidative stress under certain conditions [22].

Since UCPs are involved in the pathophysiology of glucose-related ROS cell damage, it is reasonable to hypothesize that the UCPs variants could be related to the susceptibility of DR. Yet, inconsistent results have been reported on the association analysis of UCPs variants with the risk of DR [26–28]. Herein, we aimed to conduct a meta-analysis to clarify the association of different UCPs variants with the susceptibility of DR.

## Methods

### Study design

The protocol of this meta-analysis has been registered in the international prospective register of systematic reviews (PROSPERO protocol CRD42020173510; available at https://www.crd.york.ac.uk/prospero/).

### Searching strategies and selection criteria

Three databases, including Medline Ovid, Embase Ovid and CENTRAL, were applied in the literature search for the potential studies. The eligible studies related to the susceptibility of DR and UCPs variants would be included in this meta-analysis. The following terms were used in this search: “diabetic retinopathy”, “uncoupling protein”, and “polymorphisms OR variants”. The detail search strategies and results were shown in *Supplementary document*.

Literature language was not limited to English. For languages other than Chinese and English, Google Translate (http://translate.google.com/) was used to translate the full text. The bibliographies of the screened articles have been carefully browsed to identify the omitted relevant studies.

The inclusion criteria included: 1) studies on the analysis of the association of UCPs variants with DR; 2) the recruited participants were independent and unrelated to each other; 3) sufficient genotype data for the calculation of odd ratio (OR) with 95% confidence interval (C.I.); 4) participants diagnosed with diabetes without retinopathy (DWR) would be served as the control subjects for the Hardy-Weinberg equilibrium (HWE) analysis or the data provided should be able to calculate HWE of the control group; and 5) the type of diabetes in the participants was clearly provided, including type-1 and type-2 DM. The exclusion criteria included: 1) the genotype distributions of the control subjects did not follow HWE (*P*_*HWE*_ < 0.05); and 2) the variants reported only by one study would not be included in this analysis.

### Data extraction

Two researchers (X.L. and Z.J.) independently extracted and assessed the full-text reports for all potentially eligible studies. The included studies were evaluated by the Newcastle-Ottawa quality assessment scale (NOS). The extracted items include: first author, year of publication, region of study, ethnicity, number of cases and controls, diagnostic criteria, allele or genotype frequency, Hardy-Weinberg equilibrium (HWE) status, and genotyping method. If there was any disagreement regarding to the eligibility, scores of NOS and extracted items, the judicator (Z.W.) would make the final decision. If any full-text reports have been rejected, the reasons for the rejection would be given. The data extraction form included the following data: 1) the first author and the year of publication; 2) the country and the ethnicity of the studied subjects; 3) the methodology of genotyping; 4) the methodology of DR diagnosis; 5) *P*-value of HWE in the control group; 6) The genotypic count of each variant in the patient and control groups.

Non-proliferative diabetic retinopathy (NPDR) and proliferative diabetic retinopathy (PDR) are regarded as different stages of DR, which shows different pathology and pathophysiology. This meta-analysis evaluated three types of case groups: (1) DR, (2) only PDR, and (3) combined NPDR, PDR and DR.

### Statistical analysis

A publicly available program (https://ihg.gsf.de/cgi-bin/hw/hwa1.pl) was used to estimate the HWE of the included studies. HWE in the control subjects was evaluated by χ^2^ test, and *P* < 0.05 was considered as deviation from HWE.

The association of UCPs variants with DR was evaluated by five genetic models, including the allelic (reference allele versus variant allele), homozygous (homozygous reference genotype versus homozygous variant genotype), heterozygous (homozygous reference genotype versus heterozygous genotype), dominant (homozygous reference genotype versus homozygous variant and heterozygous genotypes), and recessive models (homozygous reference and heterozygous genotypes versus homozygous variant genotype). Subgroup analyses were also conducted based on the stage of DR and the ethnicity.

Heterogeneity was examined by the Q statistic (significance defined as *P* < 0.1) and the I^2^ statistic (significant inconsistency defined as I^2^ > 50%) [29]. If heterogeneity test showed significance (*P* < 0.1 or I^2^ > 50%), the random-effect model was selected to measure the pooled effect value (DerSimonian and Laird method) [30]; otherwise, the fixed-effect model was applied (Mantel-Haenszel method) [31]. The pooled odds ratio (OR) with 95% confidence intervals (C.I.) was calculated to measure the strength of association between the UCPs variants and DR, which was assessed by the Z test (significance defined as *P* < 0.05). Sensitivity analysis was used to measure the stability of the results by excluding one study at a time when there were more than two studies. Egger’s test was used to quantitatively evaluate the potential publication bias. All statistical analysis was calculated by the STATA software (version 14.0; STATA Corporation, College Station, TX).

## Results

### Studies characteristics

**Fig. 1:**
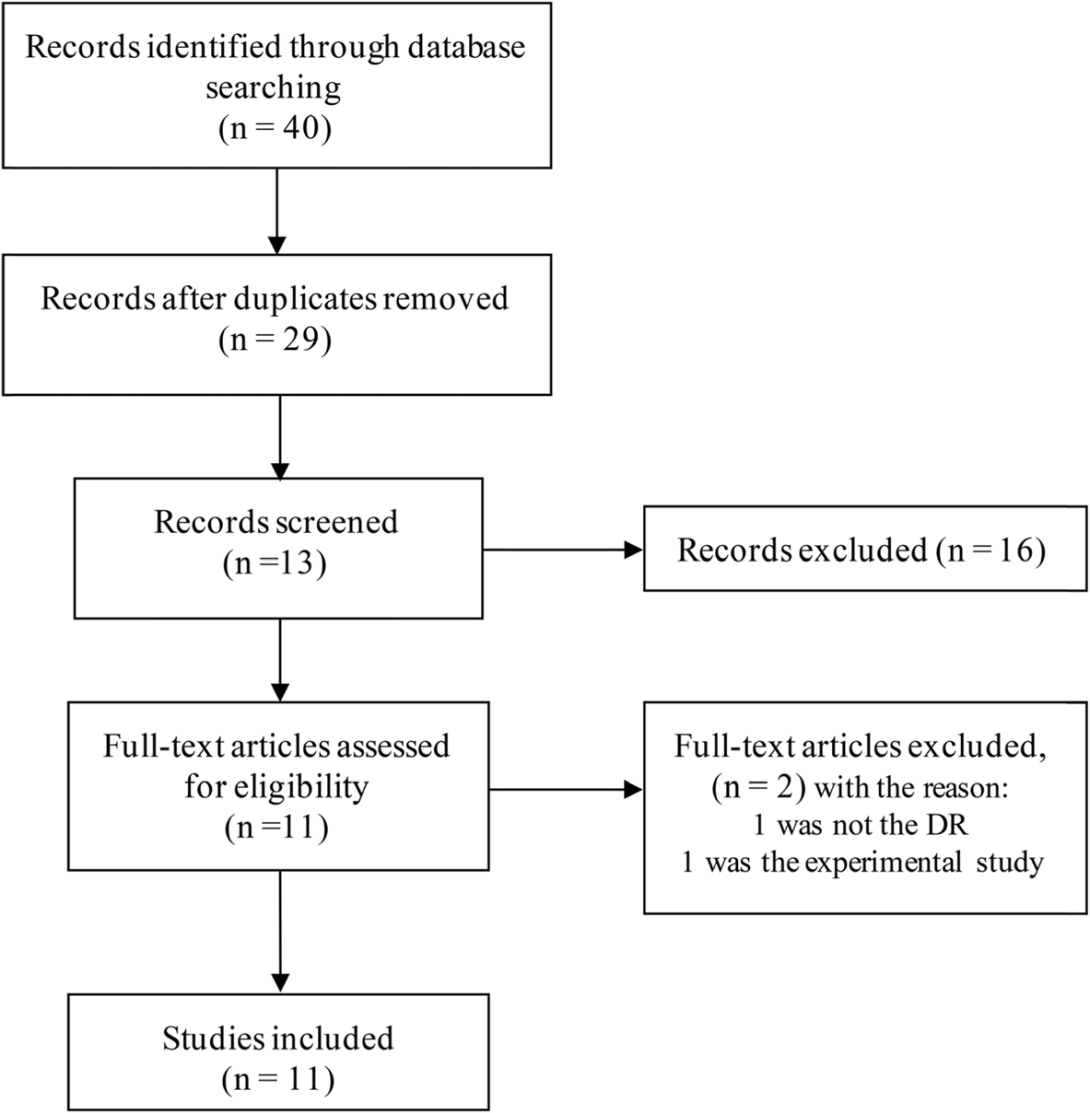
Flow chart for the inclusion and exclusion of the studies in this meta-analysis. n = number

Forty studies were resulted and retrieved from the literature search in the 3 databases. After screening on the abstracts and full-text reports, 11 studies met with the inclusion criteria (Brondani et al. 2012; Jin et al. 2017; Jin et al. 2020; Montesanto et al. 2018; Rudofsky et al. 2007; Shen et al. 2014; Zhang et al. 2014; Zhou et al. 2018; Zietz et al. 2006) [20, 26, 27, 32–37]. Jin et al. 2020 is comprised of 2 datasets, of which dataset 1 has been reported in Jin et al. 2017 [34, 35]. For Jin et al. 2020 and Montesanto et al. 2018, only the allelic data was able to be extracted [35, 36]. Therefore, dataset 2 of Jin et al. 2020 as the data for Jin 2020 and Montesanto et al. 2018 were used to calculate the pooled effect in the allelic model. The quality of the included studies was evaluated by NOS, ranging from 5 to 8, and the overall quality was moderate. The flow chart of the study selection was presented in Fig. 1.

A total of 5 UCPs variants, *UCP1* rs1800592, *UCP2* rs659366, *UCP2* rs660339 (p.A55V), *UCP2* 45-bp Ins/Del and *UCP2* rs1800849, were identified from the literature search (Table 1 and Supplementary Table 1). Yet, only rs1800592 and rs659366 were subjected to further analysis as they were reported in more than 3 studies. Eleven studies for rs1800592 and rs659366 from 4 countries, including China (*n* = 5), Brazil (*n* = 2), Germany (*n* = 3), Brazil (*n* = 2) and Italy (*n* = 1), were included. The extracted data (except for Jin et al. 2020 and Montesanto et al. 2018), HWE and minor allele frequency (MAF) were listed in Table 2. For Montesanto et al. 2018, the actual *P*_*HWE*_ value was not presented but mentioned all variants with *P*_*HWE*_ > 0.05. In addition, Jin et al. 2020 only presented HWE and MAF of the two datasets (rs1800592: MAF = 0.499, *P*_*HWE*_ > 0.999 and rs659366: MAF = 0.437, *P*_*HWE*_ = 0.640).

**Table 1:** Characteristics of the included studies

Variant	Study	Country	Ethnicity	***n*** (cases/control)	Source of controls	Type of diabetes	Genotyping methods	DMC	Method of DR acertainment	NOS
*UCP1* rs1800592	Jin 2017	China	Han Chinese	1875 (530/1345)	Population-based study	T2DM	MassARRAY	DR	Direct ophthalmoscopy, fundus fluorescein angiography and OCT	7
	Jin 2020	China	Han Chinese	1235 (134/1101)	Hospital-based case-control	T2DM	Affymetrix Genome-Wide Human SNP Array 6.0	DR	Direct ophthalmoscopy, fundus fluorescein angiography and OCT	7
	Montesanto 2018	Italy	European	940 (435/505)	Population-based study	T2DM	MassARRAY	DR	Fundoscopy through dilated pupils and/or fluorescein angiography	6
	Rudofsky 2007	Germany	Caucasian	517 (128/389)	Hospital-based case-control	T2DM	PCR-RFLPs	DR	Fundoscopy, indirect ophthalmoscopy and fundus fluorescein angiography	8
	Zhang 2014	China	Han Chinese	792 (488/344)	Hospital-based case-control	T2DM	PCR-LDR	NPDR, PDR	Fundoscopy and fundus fluorescein angiography	8
	Zietz 2006	Germany	Caucasian	509 (194/315)	Population-based study	T2DM	PCR-RFLPs	NPDR, PDR	Fundus photograpy	5
	Brondani 2012	Brazil	European	257 (154/103)	Hospital-based case-control	T1DM	PCR-RFLPs	DR	Fundoscopy through dilated pupils	6
	Rudofsky 2006	Germany	Caucasian	227 (64/163)	Hospital-based case-control	T1DM	PCR-RFLPs	DR	Ophthalmoscopic examination	7
*UCP2* rs659366	Crispim 2010	Brazil	European	501 (242/259) 196 (85/111)	Hospital-based case-control	T1DM, T2DM	PCR-RFLPs	PDR	Fundoscopy through dilated pupils	7
	Jin 2017	China	Han Chinese	1875 (530/1344)	Population-based study	T2DM	MassARRAY	DR	Direct ophthalmoscopy, fundus fluorescein angiography and OCT	7
	Jin 2020	China	Han Chinese	1235 (134/1101)	Hospital-based case-control	T2DM	Affymetrix Genome-Wide Human SNP Array 6.0	DR	Direct ophthalmoscopy, fundus fluorescein angiography and OCT	7
	Rudofsky 2007	Germany	Caucasian	645 (128/517)	Hospital-based case-control	T2DM	PCR-RFLPs	DR	Fundoscopy, indirect ophthalmoscopy and fundus fluorescein angiography	8
	Shen 2014	China	Han Chinese	472 (317/155)	Hospital-based case-control	T2DM	ABI 3100 genetic analyzer	NPDR, PDR	Fundoscopy through dilated pupils	5
	Zhou 2018	China	Han Chinese	408 (209/199)	Hospital-based case-control	T2DM	ABI 3730 genetic analyzer	NPDR, PDR	Fundoscopy and fundus photograpy	6
	Rudofsky 2006	Germany	Caucasian	227 (64/163)	Hospital-based case-control	T1DM	PCR-RFLPs	DR	Ophthalmoscopic examination	7

*UCP* uncoupling protein, *T1DM* type 1 diabetes mellitus, *T2DM* type 2 diabetes mellitus, *DMC* diabetes mellitus complications, *DR* diabetic retinopathy, *PDR* proliferative diabetic retinopathy, *NPDR* non-proliferative diabetic retinopathy, *NOS* Newcastle-Ottawa quality assessment scale, *n*: number

**Table 2:** Extracted genotype data from the included studies

Variant	Controls (***n***)	Cases (***n***)	Study	Cases					Controls					***P***_***HWE***_	MAF
*UCP1* rs1800592				GG	GA	AA	G	A	GG	GA	AA	G	A		
	DWR (2260)	DR (1386)	Jin 2017	140	251	139	531	529	338	669	338	1345	1345	0.85	50%
			Rudofsky 2007	5	63	60	73	183	18	173	198	209	569	0.01	27%
			Zhang 2014	122	217	105	461	427	79	161	94	319	349	0.53	50%
			Zietz 2006	8	72	114	88	300	15	117	183	147	483	0.50	23%
	DWR (649)	NPDR (366)	Zhang 2014	57	110	60	224	230	79	161	94	319	349	0.53	48%
			Zietz 2006	6	49	84	61	217	15	117	183	147	483	0.50	23%
	DWR (649)	PDR (272)	Zhang 2014	65	107	45	237	197	79	161	94	319	349	0.53	50%
			Zietz 2006	2	23	30	27	83	15	117	183	147	483	0.50	24%
*UCP2* rs659366				AA	AG	GG	A	G	AA	AG	GG	A	G		
	DWR (2244)	DR (1236)	Jin 2017	68	261	201	397	663	177	654	513	1008	1680	0.16	37%
			Rudofsky 2007	17	62	49	96	160	49	186	154	284	494	0.54	37%
			Shen 2014	59	144	102	262	348	38	73	38	149	149	0.81	45%
			Zhou 2018	57	122	30	236	182	33	110	56	176	222	0.09	50%
	DWR (348)	NPDR (194)	Shen 2014	23	54	25	100	104	38	73	38	149	149	0.81	50%
			Zhou 2018	25	61	6	111	73	33	110	56	176	222	0.09	49%
	DWR (606)	PDR (560)	Crispim 2010	45	131	64	221	259	30	116	112	176	340	1.00	40%
			Shen 2014	36	90	77	162	244	38	73	38	149	149	0.81	44%
			Zhou 2018	32	61	24	125	109	33	110	56	176	222	0.09	48%

*DWR* diabetes without retinopathy, *DR* diabetic retinopathy, *PDR* proliferative diabetic retinopathy, *NPDR* non-proliferative diabetic retinopathy, *HWE* Hardy-Weinberg equilibrium, *MAF* minor allele frequency, *n*: number

### Data analysis

**Fig. 2:**
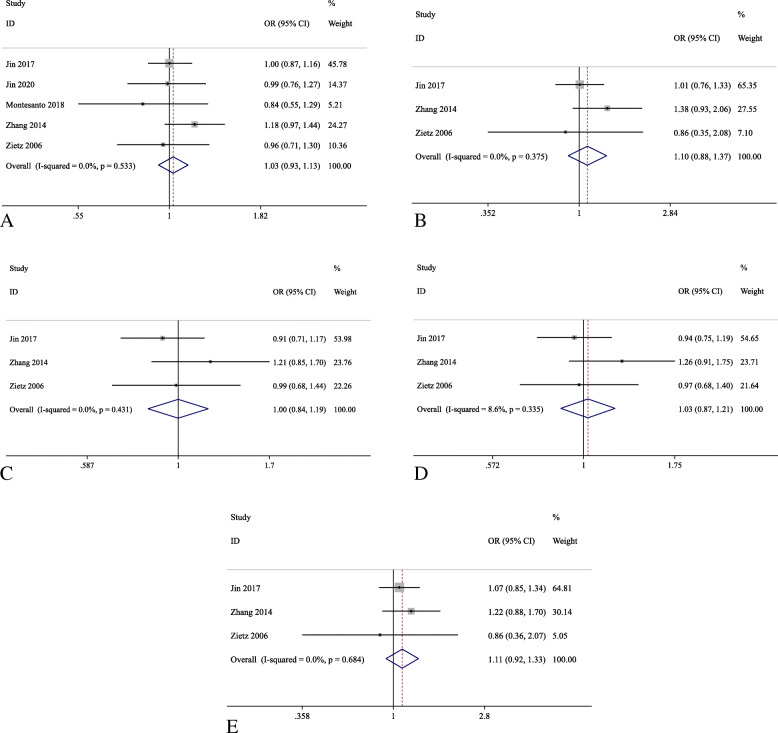
Forest plots for the association analysis between the *UCP1* rs1800592 variant and diabetic retinopathy in type-2 diabetes mellitus patients in five genetic models. Reference allele: A; variant allele: G. **a** Allelic (G vs A); **b** Homozygous (GG vs AA); **c** Heterozygous (AG *V. AA*); **d** Dominant (GG + AG vs AA); **e** Recessive (GG vs AG + AA) models

**Fig. 3:**
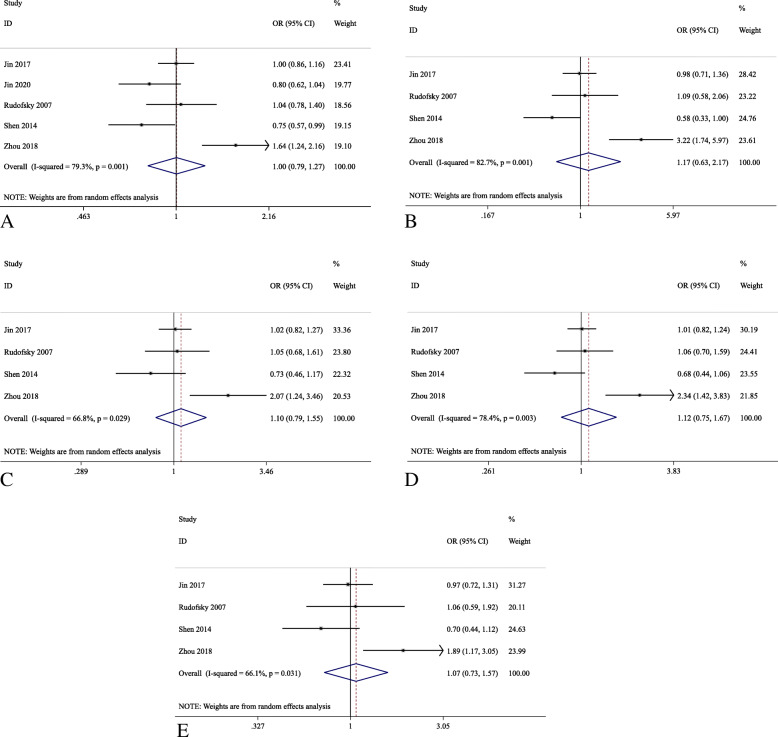
Forest plots for the association analysis of *UCP2* rs659366 variant with diabetic retinopathy in type-2 diabetes mellitus patients in five genetic models Reference allele: G; variant allele: A. **a** Allelic (A vs G); **b** Homozygous (AA vs GG); **c** Heterozygous (GA vs GG); **d** Dominant (AA+GA vs GG); **e** Recessive (AA vs GA + GG) models.

**Fig. 4:**
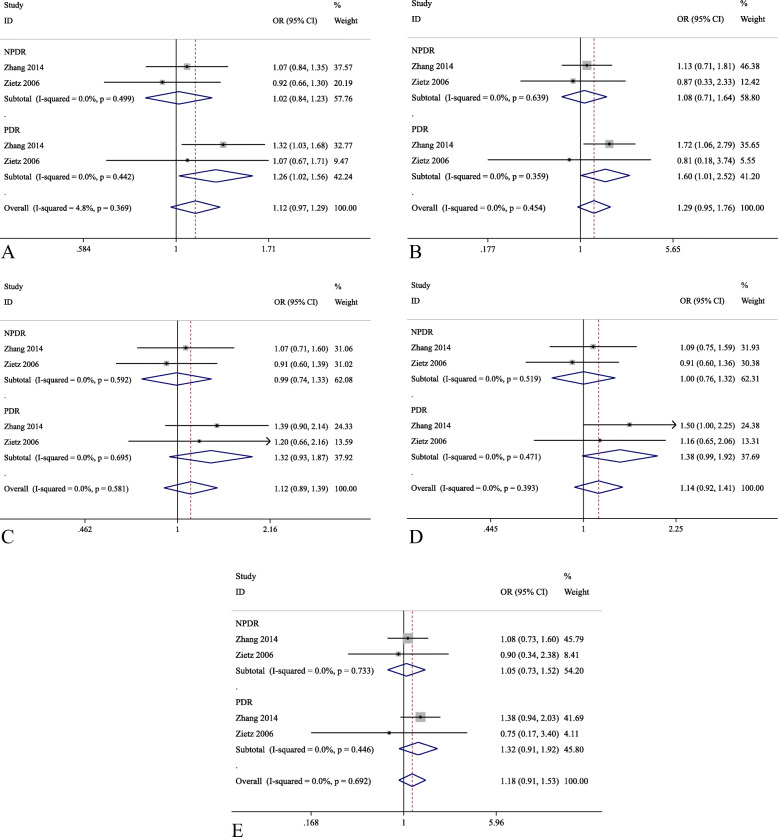
Forest plots for the subgroup analysis of *UCP1* rs1800592 variant with the stage of diabetic retinopathy in type-2 diabetes mellitus patients in five genetic models. Reference allele: A; variant allele: G. **a** Allelic (G vs A); **b** Homozygous (GG vs AA); **c** Heterozygous (AG *V. AA*); **d** Dominant (GG + AG vs AA); **e** Recessive (GG vs AG + AA) models

Five studies were identified for the investigation of *UCP1* rs1800592, among which Rudofsky et al. 2007 was not included in the pooled effects analysis as its *P*_*HWE*_ in the control subjects was less than 0.05 [26]. Only 2 studies, Brondani et al. 2012 and Crispim et al. 2010, included the patients with type-1 DM [20, 28]. Hence, rs1800592 and rs659366 were further analyzed only with patients in type-2 DM. For *UCP1* rs1800592, a total of 1781 patients as cases (DR) and 3610 patients as control (DWR) was used for the meta-analysis in the allelic model, and the number of cases and controls in other models were 1212 and 2004, respectively. Similarly, for *UCP2* rs659366, the number of cases and controls in the allelic model were 1318 and 3316, respectively, and 1318 patients as case and 3316 patients as control in other models. The pooled effect analysis of *UCP1* rs1800592 showed no significant association in type-2 DM patients for all five genetic models (Table 3 and Fig. 2). Similarly, for *UCP2* rs659366, the pooled effect analysis showed no statistically significant association with DR in type-2 DM patients for all five genetic models (Table 3 and Fig. 3). In the subgroup analyses, *UCP1* rs1800592 showed statistically significant association with PDR in type-2 DM patients for the allelic (G allele versus A allele: OR = 1.26, 95% C.I.: 1.02–1.56, *P* = 0.035), homozygous model (GG versus AA: OR = 1.60, 95% C.I.: 1.01–2.52, *P* = 0.044), but not for the heterozygous, dominant and recessive models. (*P* > 0.05; Table 4 and Fig. 4). However, for other subgroup analyses, no statistically significant association was found in the stage of DR and ethnicity (Supplementary figure 1, 2, 3).

**Table 3:** Pooled effect on the association of UCPs variants with DR in type-2 DM patients

Comparison	Variant	Model	Effect	Pooled OR (95% C.I.)	Z	***P***_**z**_	I^**2**^	***P*** for heterogeneity
DR vs DWR	*UCP1* rs1800592	Allelic (G vs A)	Fixed	1.03 (0.93, 1.13)	0.51	0.609	0.0%	0.533
		Homozygous (GG vs AA)	Fixed	1.10 (0.88, 1.37)	0.85	0.398	0.0%	0.375
		Heterozygous (AG vs AA)	Fixed	1.00 (0.84, 1.19)	0.01	0.992	0.0%	0.431
		Dominant (GG + AG vs AA)	Fixed	1.03 (0.87, 1.21)	0.31	0.760	8.6%	0.335
		Recessive (GG vs AG + AA)	Fixed	1.11 (0.92, 1.33)	1.07	0.284	0.0%	0.684
DR vs DWR	*UCP2* rs659366	Allelic (A vs G)	Random	1.00 (0.79, 1.27)	0.02	0.983	79.3%	0.001
		Homozygous (AA vs GG)	Random	1.17 (0.63, 2.17)	0.49	0.623	82.7%	0.001
		Heterozygous (GA vs GG)	Random	1.10 (0.79, 1.55)	0.56	0.572	66.8%	0.029
		Dominant (AA+GA vs GG)	Random	1.12 (0.75, 1.67)	0.55	0.584	78.4%	0.003
		Recessive (AA vs GA + GG)	Random	1.07 (0.73, 1.57)	0.35	0.73	66.1%	0.031

*DM* diabetes mellitus, *DWR* diabetes without retinopathy, *DR* diabetic retinopathy, *PDR* proliferative diabetic retinopathy, *NPDR* non-proliferative diabetic retinopathy, *C.I.* confident interval

**Table 4:** Subgroup analysis on subtype of DR and ethnicity

Variant	Cases vs Controls	Ethnicity	Allelic	Homozygous	Heterozygous	Dominant	Recessive
			OR (95% C.I.)	OR (95% C.I.)	OR (95% C.I.)	OR (95% C.I.)	OR (95% C.I.)
*UCP1* rs1800592			G vs A	GG vs AA	AG vs AA	GG + AG vs AA	GG vs AG + AA
	NPDR vs DWR		1.02 (0.84,1.23)	1.08 (0.71,1.64)	0.99 (0.74,1.33)	1.00 (0.76,1.32)	1.05 (0.73,1.52)
	PDR vs DWR		1.26 (1.02,1.56)*	1.60 (1.01,2.52)*	1.32 (0.93, 1.87)	1.38 (0.99,1.92)	1.32 (0.91,1.92)
*UCP2* rs659366			A vs G	AA vs GG	GA vs GG	AA+GA vs GG	AA vs GA + GG
	NPDR vs DWR		1.36 (0.69,2.67)	2.48 (0.33, 18.37)	2.33 (0.51,10.57)	2.35 (0.45, 12.43)	1.26 (0.58,2.75)
	PDR vs DWR		1.17 (0.67,2.05)	1.40 (0.46,4.24)	1.17 (0.57,2.40)	1.22 (0.54,2.77)	1.28 (0.63,2.56)
*UCP1* rs1800592			G vs A	GG vs AA	AG vs AA	GG + AG vs AA	GG vs AG + AA
	DR vs DWR	Chinese	1.05 (0.94, 1.16)	1.12 (0.89,1.40)	1.00 (0.82,1.22)	1.04 (0.86,1.26)	1.12 (0.93,1.35)
		Total	1.03 (0.93, 1.13)	1.10 (0.88,1.37)	1.00 (0.84,1.19)	1.03 (0.87,1.21)	1.11 (0.92, 1.33)
*UCP2* rs659366			A vs G	AA vs GG	GA vs GG	AA+GA vs GG	AA vs GA + GG
	DR vs DWR	Chinese	0.99 (0.74, 1.34)	1.20 (0.52,2.76)	1.13 (0.70,1.83)	1.15 (0.65,2.03)	1.08 (0.65,1.77)
		Total	1.00 (0.79,1.27)	1.17 (0.63,2.17)	1.10 (0.79,1.55)	1.12 (0.75,1.67)	1.07 (0.73,1.57)

*P < 0.05, *DWR* diabetes without retinopathy, *DR* diabetic retinopathy, *PDR* proliferative diabetic retinopathy, *NPDR* non-proliferative diabetic retinopathy, *C.I.* confident interval

### Evaluation of publication bias and sensitivity analyses

**Fig. 5:**
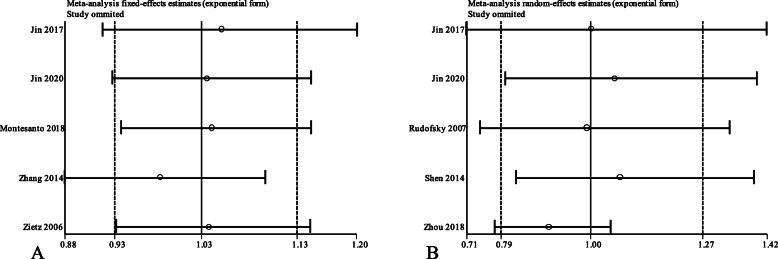
Sensitivity analysis of *UCP1* rs1800592 and *UCP2* rs659366 variants in allelic model. **a** rs1800592 (G vs A); **b** rs659366 (A vs G)

The Egger’s test, which was used to quantitatively measure the publication bias, showed no statistically significant publication bias (*P* > 0.05; Table 5). The results of the sensitivity analysis showed that the pooled OR lied within the 95% C.I. of the total pooled OR (Fig. 5).

**Table 5:** Evaluation of publication bias by Egger’s test

Variant	Cases vs Controls	Allelic	Homozygous	Heterozygous	Dominant	Recessive
*UCP1* rs1800592	DR vs DWR	0.482	0.970	0.520	0.657	0.763
*UCP2* rs659366	DR vs DWR	0.944	0.687	0.710	0.702	0.770

α = 0.1

## Discussion

DR is one of the most common microvascular complications in DM patients. The hyperglycemia-induced ROS is considered as one of the initial and major pathways causing the damage to the endothelial cells. The UCPs are anion carrier proteins in mitochondrial inner membrane. UCPs function to reduce mitochondrial ROS, especially hyperglycemia-induced oxidative stress, and protect endothelial cells from oxidative stress by balancing the proton motive force across the mitochondrial inner membrane [22]. Therefore, UCPs could possibly participate in the development and pathogenesis of DR.

This meta-analysis verified the association of the reported UCPs variants with the susceptibility of DR. Our results showed that *UCP1* rs1800592 variant was not significantly associated with DR in type-2 DM patients in the pooled effects analysis (Table 3 and Fig. 2); yet, in the subgroup analysis, *UCP1* rs1800592 was significantly associated with PDR in type-2 DM patients in the allelic and homozygous models (Table 4 and Fig. 4). The patients carrying allele G of *UCP1* rs1800592 variant have 26% higher risk developing PDR than those carrying allele A. A previous study demonstrated that the carriers of rs1800592 GG genotype exhibited higher *UCP1* gene expression than those with AA genotype in the retina samples [20]. This could suggest that higher *UCP1* expression in retina by allele G of rs1800592 variant could be associated with higher susceptibility of PDR. Conversely, *UCP1* expression was lower in carriers of GG genotype than those with AA genotype in intraperitoneal adipose cells, indicated the tissue-specific effect of rs1800592 on UCP1 expression activity [38]. Moreover, allele G of *UCP1* rs1800592 also showed elevated expression of MnSOD2 gene, which is another major scavenger for mitochondrial ROS [20, 39]. Our discovery was resulted from 2 reported studies, and our approach is similar to that from our previous studies [40–42]. Nevertheless, further studies with larger cohorts in different populations are needed to verify its association with PDR.

UCP2 is the most widely distributed uncoupling protein and most frequently studied in DM and DR, and itis associated with the increased oxidative stress and negatively regulates the insulin secretion [43, 44]. Total 4 *UCP2* variants, *UCP2* rs659366, *UCP2* rs660339 (p.A55V), *UCP2* 45-bp Ins/Del and *UCP2* rs1800849, were reported in the association analysis with DR; however, only *UCP2* rs659366 variant comprised enough studies for the meta-analysis, and other *UCP2* variants have not been further analyzed in this study. *UCP2* rs659366 has been reported to be associated with type-2 DM [45]; however, in this meta-analysis, we demonstrated that *UCP2* rs659366 variant showed no pooled association with DR in the type-2 DM patients (Table 3 and Fig. 3). The elevation of UCP2 expression could be induced by high glucose treatment in epithelial cell of human vein, and the A allele of *UCP2* rs659366 increases promoter activity as compared to the G allele, which can be exacerbated under hyperglycemic condition to exert a protective effect [46]. The negative association of UCP2 rs659366 variant with DR in this meta-analysis might indicated that *UCP2* gene variation may not be contributed to the development of DR. Nevertheless, it is of worth to note that, in the F-SNP database analyses, *UCP2* rs660339 is strongly linked with *UCP2* rs659366, and partially linked with *UCP2* 45-bp Ins/Del variant [28]. One report showed that the haplotype of 3 different*UCP2* variants [Ins (45-bp Ins/Del), A (rs659366) and Ala (rs660339)] is associated with the decreased *UCP2* gene expression in human retina [47]. This could be an independent risk factor for PDR in both type-1 and 2 DM patients [28]. Additional association studies are necessary in order to confirm the association of all 4 *UCP2* variants with DR in different ethnic groups.

We conducted the subgroup analyses on ethnicity in this meta-analysis. There was no significant association in different ethnic group, which could be due to the limited and sample sizes after stratification. Thus, the ethnicity-specific effects of these variants need to be determined with larger sample sizes in additional cohort studies.

There are several limitations in this meta-analysis. First, the number of reported studies for each UCPs variant was still limited. Second, the lack of original clinical information would be difficult to adjust the relevant variables, such as duration of diabetes, medications and other chronic diseases.

## Conclusions

In summary, our meta-analysis revealed no significant pooled association of *UCP1* rs1800592 and *UCP2* rs659366 with DR in DM patients; yet allele G of *UCP1* rs1800592 variant could be associated with the increased risk of PDR in type-2 DM patients. Our results suggest that *UCP1* rs1800592 variant could be clinically applied as a genetic marker for PDR prediction and risk analysis in DM clinics.

## Supplementary Information

**Additional file 1.**

**Additional file 2.**

**Additional file 3.**

**Additional file 4: Table S1**. Characteristics of other UCPs variants. **Table S2**. Analysis of the included studies by Newcastle-Ottawa quality assessment scale. **Figure S1**. Subgroup analysis of *UCP1* rs1800592 by ethnicity. **Figure S2**. Subgroup analysis of *UCP2* rs659366 by ethnicity. **Figure S3**. Subgroup analysis of *UCP2* rs659366 by stage of DR. **Figure S4**. Sensitivity analyses of *UCP1* rs1800592 in all genetic models. **Figure S5**. Sensitivity analyses of *UCP2* rs659366 in all genetic models

## Data Availability

All the data supporting our findings is contained within the manuscript.

## Abbreviations

UCPs: Uncoupling proteins
DR: Diabetic retinopathy
DM: Diabetes mellites
OR: Odds ratios
PDR: Proliferative diabetic retinopathy
C.I.: Confidence interval
DWR: Diabetes without retinopathy
HWE: Hardy-Weinberg equilibrium
NOS: Newcastle-Ottawa quality assessment scale
MAF: Minor allele frequency
