# Supplementary material for: Association of UCP1 and UCP2 variants with diabetic retinopathy susceptibility in type-2 diabetes mellitus patients: a meta-analysis
Source: BMC Ophthalmol. 2021 Feb 12;21:81. doi: 10.1186/s12886-021-01838-1 (PMC7881628; doi:10.1186/s12886-021-01838-1)

**Supplementary table 1: Characteristics of other UCPs variants.**

| Variant | Study | Country | Ethnicity | Source | Type of diabetes | Genotyping methods | DMC | Controls | Methodof DR acertainment | NOS |
| --- | --- | --- | --- | --- | --- | --- | --- | --- | --- | --- |
| *UCP2* | Crispim 2010 | Brazil | European | Hospital-based case-control | T1DM, T2DM | TaqMan | PDR | DWR | Fundoscopy through dilated pupils | 7 |
| rs660339 | Montesanto 2018 | Italy | European | Population-based study | T2DM | MassARRAY | DR | DWR | Fundoscopy through dilated pupils and/or fluorescein angiography | 6 |
|  | Shen 2014 | China | Han Chinese | Hospital-based case-control | T2DM | ABI 3100 genetic analyzer | NPDR, PDR | DWR | Fundoscopy through dilated pupils | 5 |
| *UCP2* | Crispim 2010 | Brazil | European | Hospital-based case-control | T1DM, T2DM | PCR-RFLPs | PDR | DWR | Fundoscopy through dilated pupils | 7 |
| 45-bp Ins/Del | Zhou 2018 | China | Han Chinese | Hospital-based case-control | T2DM | ABI 3730 genetic analyzer | NPDR, PDR | DWR | Fundoscopy and fundus photograpy | 6 |
| *UCP3* | Rudofsky 2007 | Germany | Caucasian | Hospital-based case-control | T2DM | PCR-RFLPs | DR | DWR | Fundoscopy, indirect ophthalmoscopy and fundus fluorescein angiography | 8 |
| rs1800849 | Rudofsky 2006 | Germany | Caucasian | Hospital-based case-control | T1DM | PCR-RFLPs | DR | DWR | Ophthalmoscopic examination | 7 |

UCP: uncoupling protein, T1DM: type 1 diabetes mellitus, T2DM: type 2 diabetes mellitus, DWR: diabetes without retinopathy, DR: diabetic retinopathy, PDR: proliferative diabetic retinopathy, NPDR: non-proliferative diabetic retinopathy, NOS: Newcastle-Ottawa quality assessment scale.

**Supplementary table 2: Analysis of the included studies by Newcastle-Ottawa quality assessment scale.**

|  |  | Brondani 2012 | Crispim 2010 | Jin 2017 | Rudofsky 2007 | Rudofsky 2006 | Shen 2014 | Zhang 2014 | Zhou 2018 | Zietz 2006 | Jin 2020 | Montesanto 2018 |
| --- | --- | --- | --- | --- | --- | --- | --- | --- | --- | --- | --- | --- |
| Selection | case definition adequate | a | a | b | a | a | b | a | a | b | b | b |
|  | Representativeness of the cases | b | b | a | a | b | b | a | b | b | b | a |
|  | Selection of Conrols | b | b | a | c | c | b | b | c | c | a | c |
|  | Definition of Controls | a | a | a | a | a | a | a | b | b | a | b |
| Comparability | Comparability | a | ab | a | ab | ab | a | ab | ab | ab | ab | ab |
| Exposure | Ascertainment of exposure | a | a | a | a | a | a | a | a | a | a | a |
|  | Same method of ascertainment for cases and controls | a | a | a | a | a | a | a | a | a | a | a |
|  | Non-Response rate | a | a | a | a | a | a | a | a | a | a | a |
|  | NOS | 6 | 7 | 7 | 8 | 7 | 5 | 8 | 6 | 5 | 7 | 6 |

**
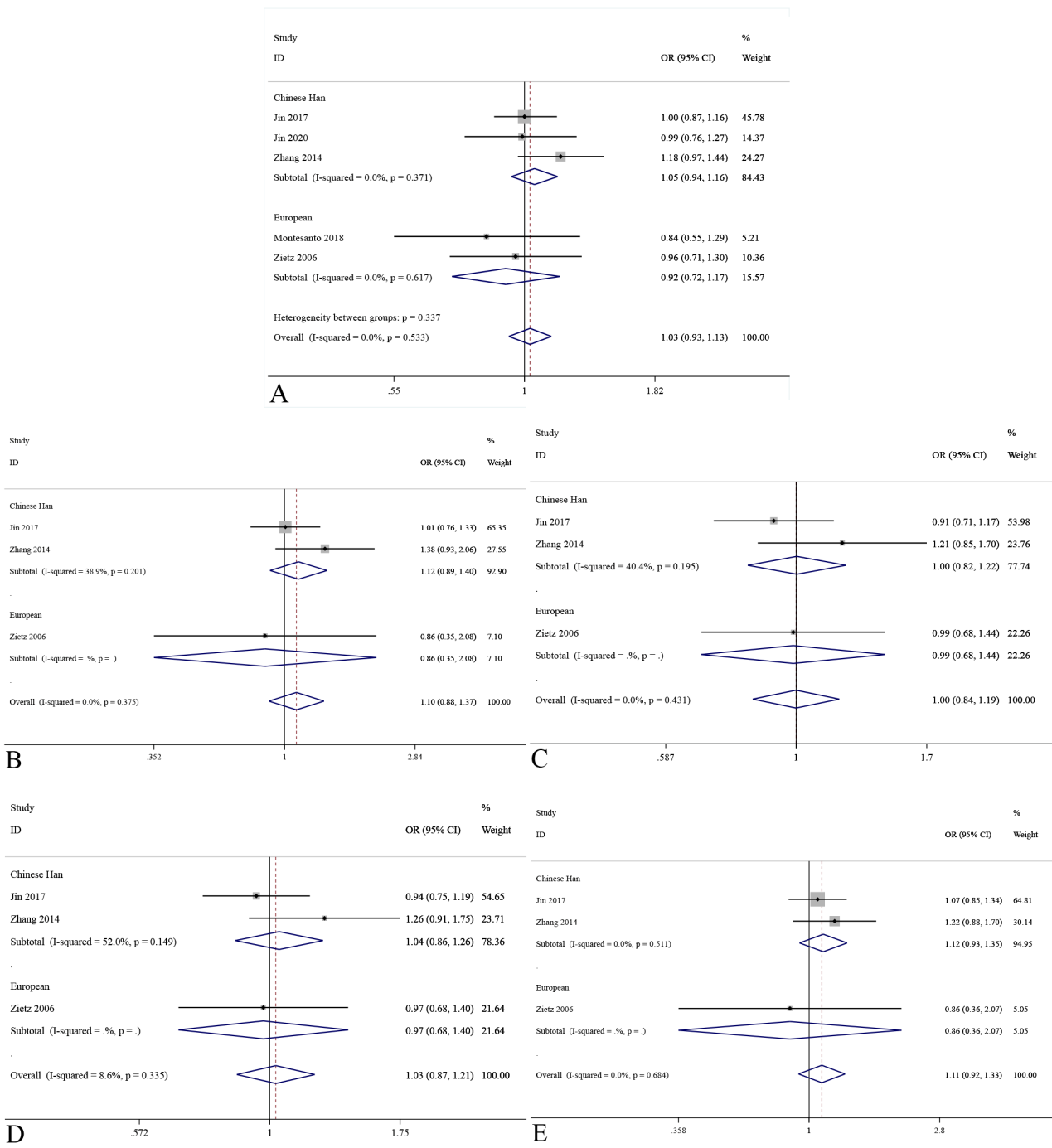
**

**Supplementary figure 1:** **Subgroup analysis of *UCP1* rs1800592 by ethnicity.**

DR vs DWR: Reference allele: A; variant allele: G. (**A**) Allelic (G vs A); (**B**) Homozygous (GG vs AA); (**C**) Heterozygous (AG vs AA); (**D**) Dominant (GG+AG vs AA); (**E**) Recessive (GG vs AG+AA) models.

**
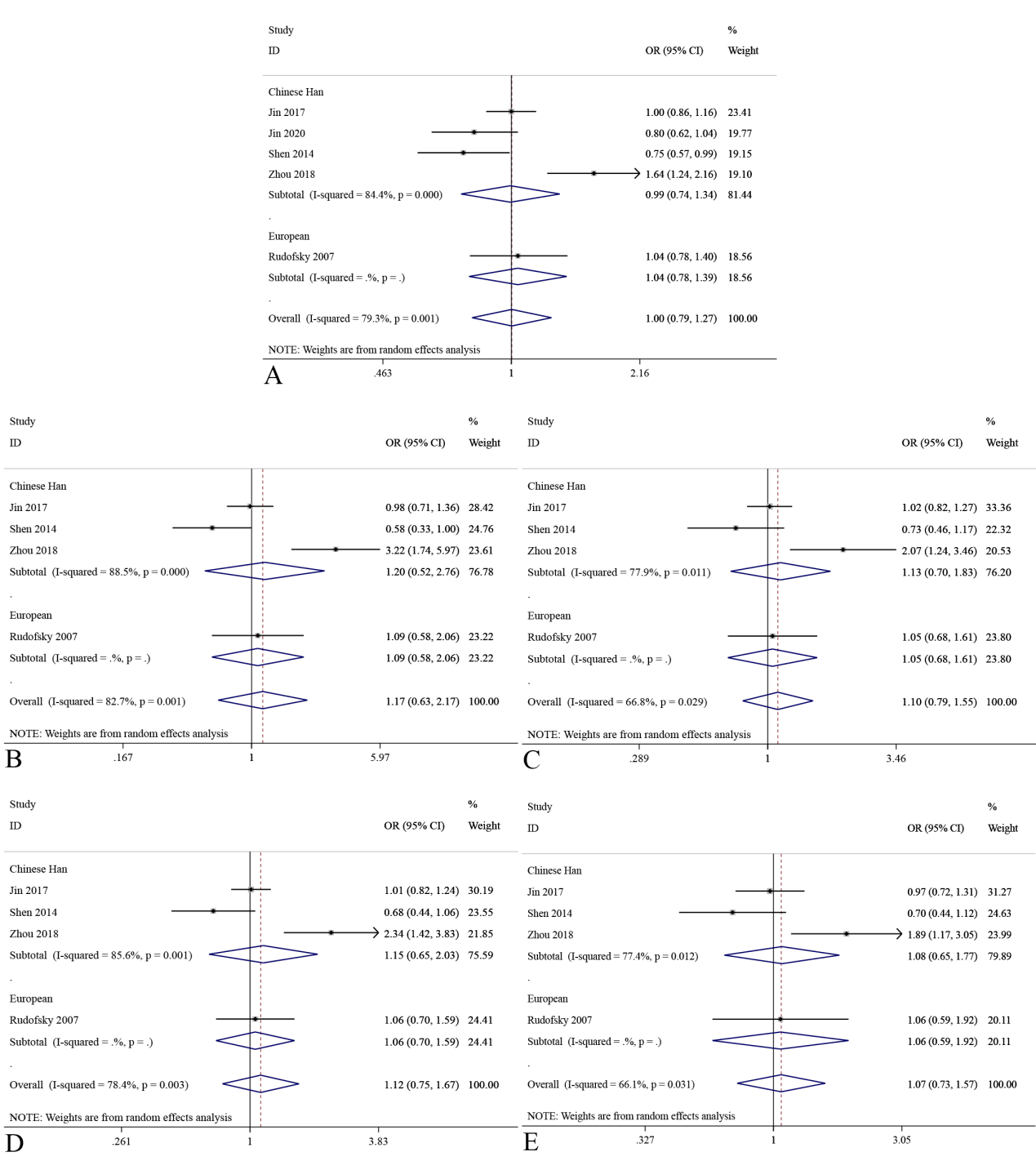
**

**Supplementary figure 2:** **Subgroup analysis of *UCP2* rs659366 by ethnicity.**

DR vs DWR: Reference allele: G; variant allele: A. (**A**) Allelic (A vs G); (**B**) Homozygous (AA vs GG); (**C**) Heterozygous (GA vs GG); (**D**) Dominant (AA+GA vs GG); (**E**) Recessive (AA vs GA+GG) models.


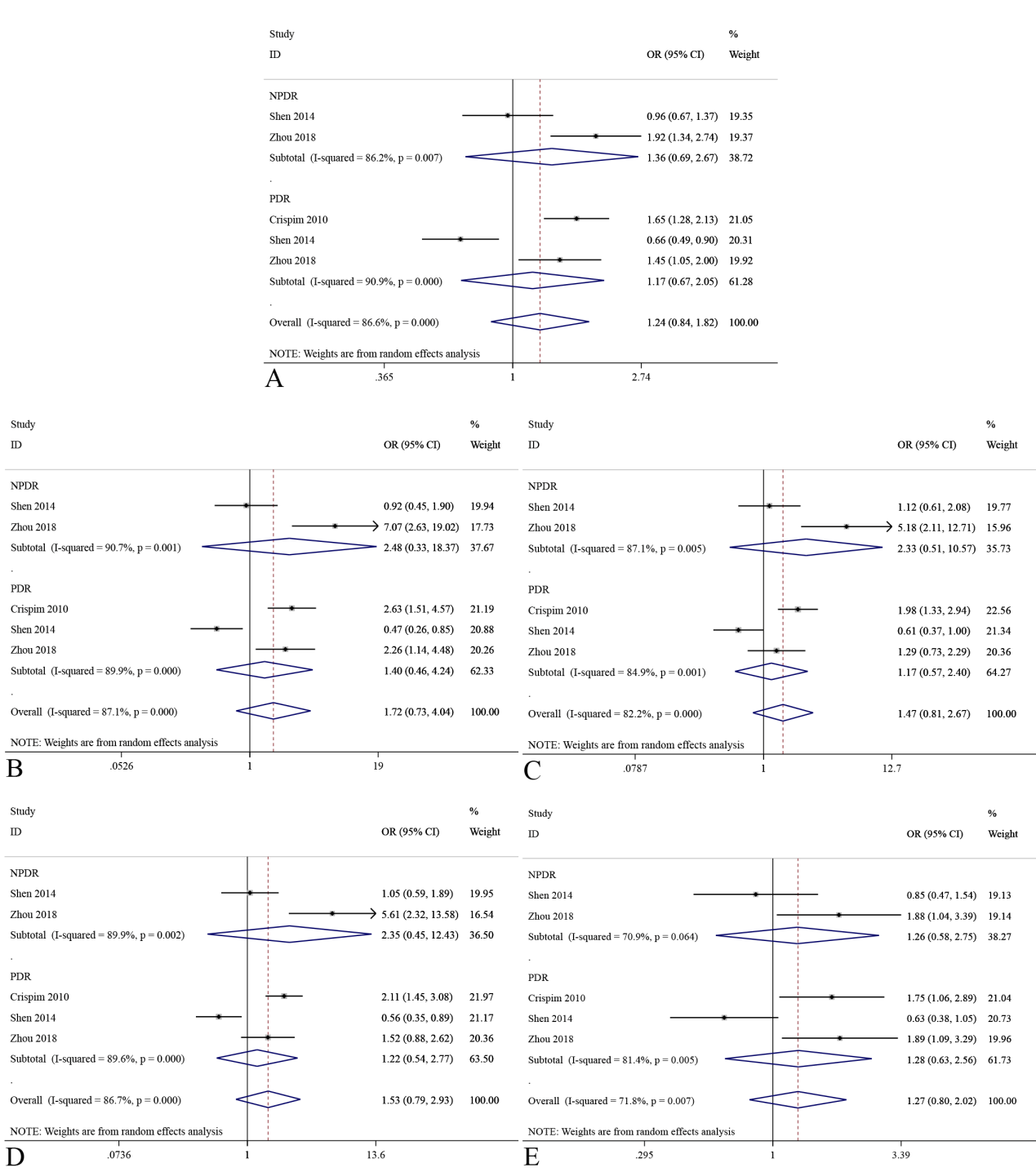


**Supplementary figure 3: Subgroup analysis of *UCP2* rs659366 by stage of DR.**

Reference allele: G; variant allele: A. (**A**) Allelic (A vs G); (**B**) Homozygous (AA vs GG); (**C**) Heterozygous (GA vs GG); (**D**) Dominant (AA+GA vs GG); (**E**) Recessive (AA vs GA+GG) models.





**Supplementary figure 4: Sensitivity analyses of *UCP1* rs1800592 in all genetic models.**

Reference allele: A; variant allele: G. (**A**) Allelic (G vs A); (**B**) Homozygous (GG vs AA); (**C**) Heterozygous (AG vs AA); (**D**) Dominant (GG+AG vs AA); (**E**) Recessive (GG vs AG+AA) models.





**Supplementary figure 5: Sensitivity analyses of *UCP2* rs659366 in all genetic models.**

Reference allele: G; variant allele: A. (**A**) Allelic (A vs G); (**B**) Homozygous (AA vs GG); (**C**) Heterozygous (GA vs GG); (**D**) Dominant (AA+GA vs GG); (**E**) Recessive (AA vs GA+GG) models.

**Detail search strategies and results**

**Medline Ovid (last search on 09/20/2020)**

1. exp Diabetic Retinopathy/
2. ((diabet$ or proliferative or non-proliferative) adj4 retinopath$).tw.
3. (diabet$ adj3 (eye$ or vision or visual$ or sight$)).tw.
4. (DR adj3 (eye$ or vision or visual$ or sight$)).tw.
5. (diabet$ adj3 maculopath$).tw.
6. or/1-5
7. exp Polymorphism, Single Nucleotide/
8. SNP$.tw.
9. Polymorphism$.tw.
10. or/7-9
11. uncoupling protein.tw.
12. uncoupling proteins.tw.
13. UCP$.tw.
14. or/11-13
15. 6 and 10 and 14

Search results


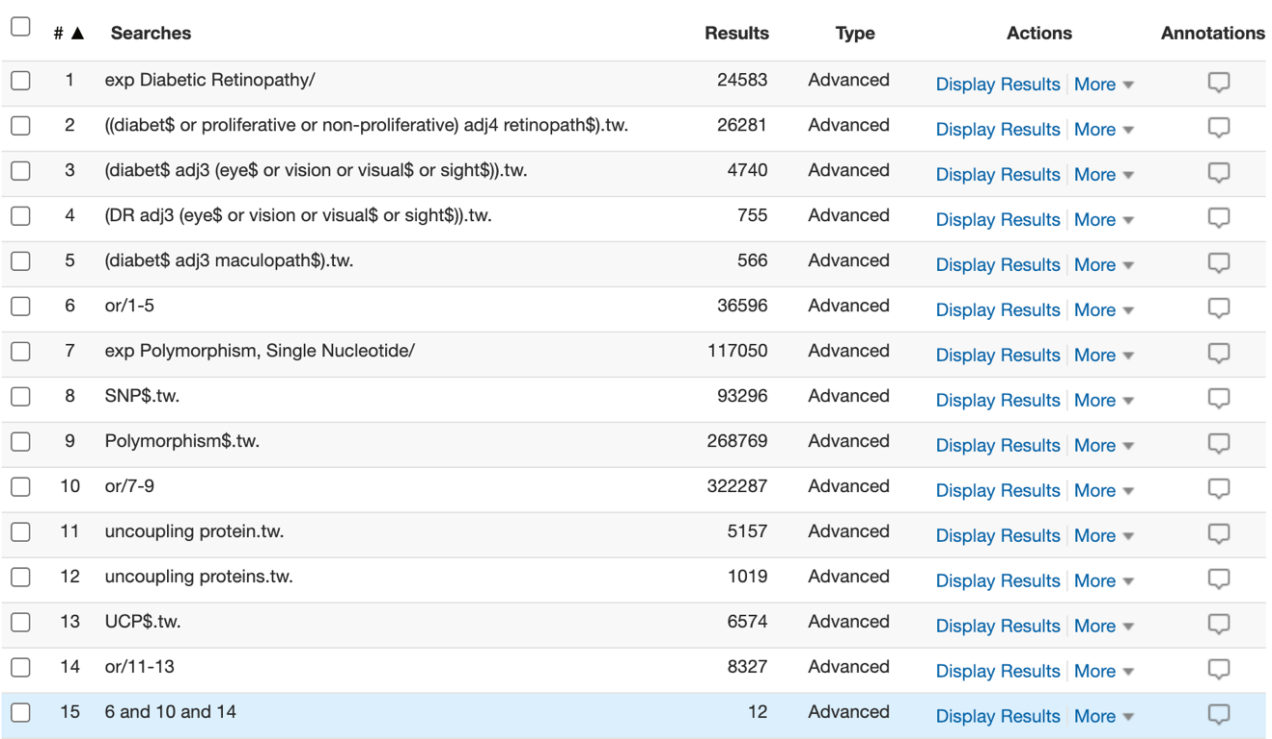


**Embase Ovid (last search on 09/20/2020)**

1. exp Diabetic Retinopathy/
2. ((diabet$ or proliferative or non-proliferative) adj4 retinopath$).tw.
3. (diabet$ adj3 (eye$ or vision or visual$ or sight$)).tw.
4. (DR adj3 (eye$ or vision or visual$ or sight$)).tw.
5. (diabet$ adj3 maculopath$).tw.
6. or/1-5
7. exp Polymorphism, Single Nucleotide/
8. SNP$.tw.
9. Polymorphism$.tw.
10. or/7-9
11. uncoupling protein.tw.
12. uncoupling proteins.tw.
13. UCP$.tw.
14. or/11-13
15. 6 and 10 and 14

Search results


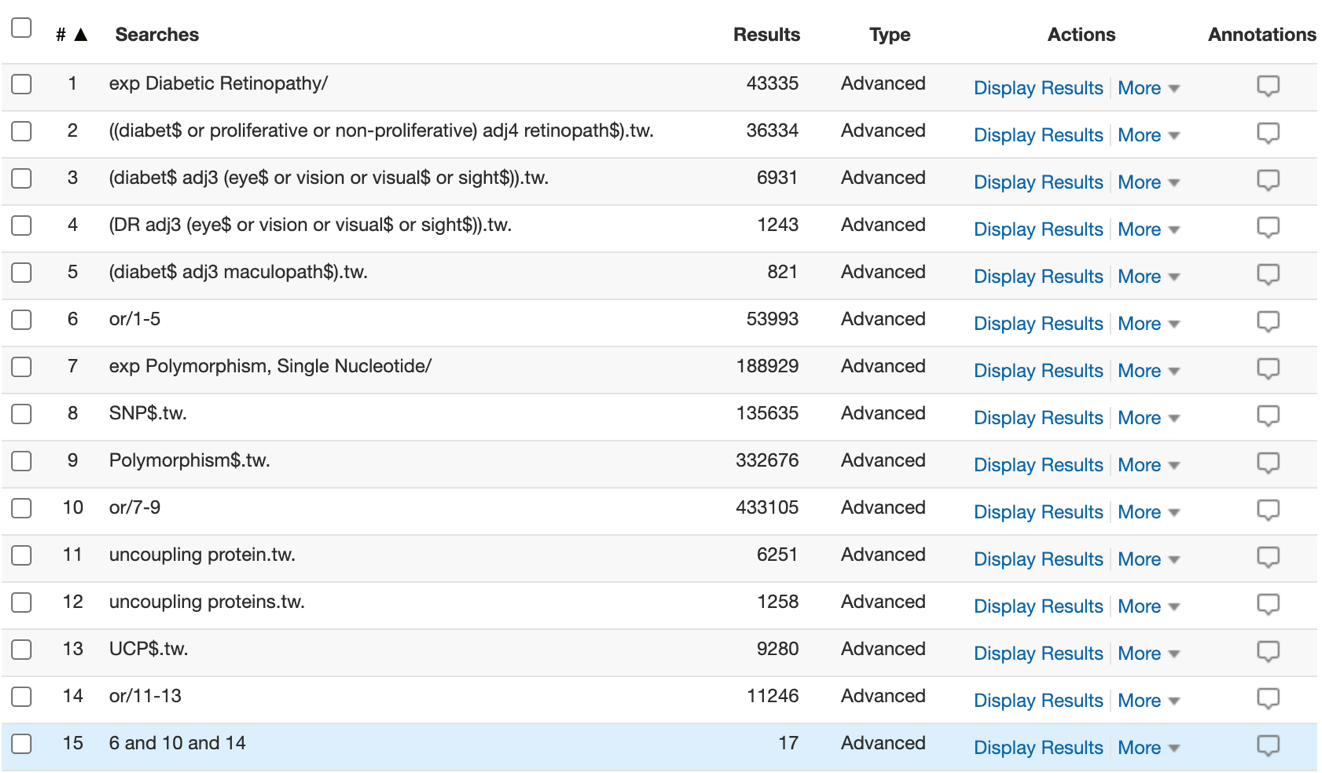


**CENTRAL_11 papers (last search on 09/20/2020)**

1. MeSH descriptor: [Diabetic Retinopathy] explode all trees
2. (diabet* or proliferat* or non-proliferat*) near4 retinopath*
3. diabet* near3 (eye* or vision or visual* or sight*)
4. DR near3 (eye* or vision or visual* or sight*)
5. diabet* near3 maculopath*
6. #1 OR #2 OR #3 OR #4 OR #5
7. MeSH descriptor: [Polymorphism, Single Nucleotide] explode all trees
8. SNP*
9. polymorphism*
10. #7 OR #8 OR #9
11. uncoupling proteins
12. uncoupling protein
13. UCP*
14. #11 OR #12 OR #13
15. #6 AND #10 AND #14

Search results


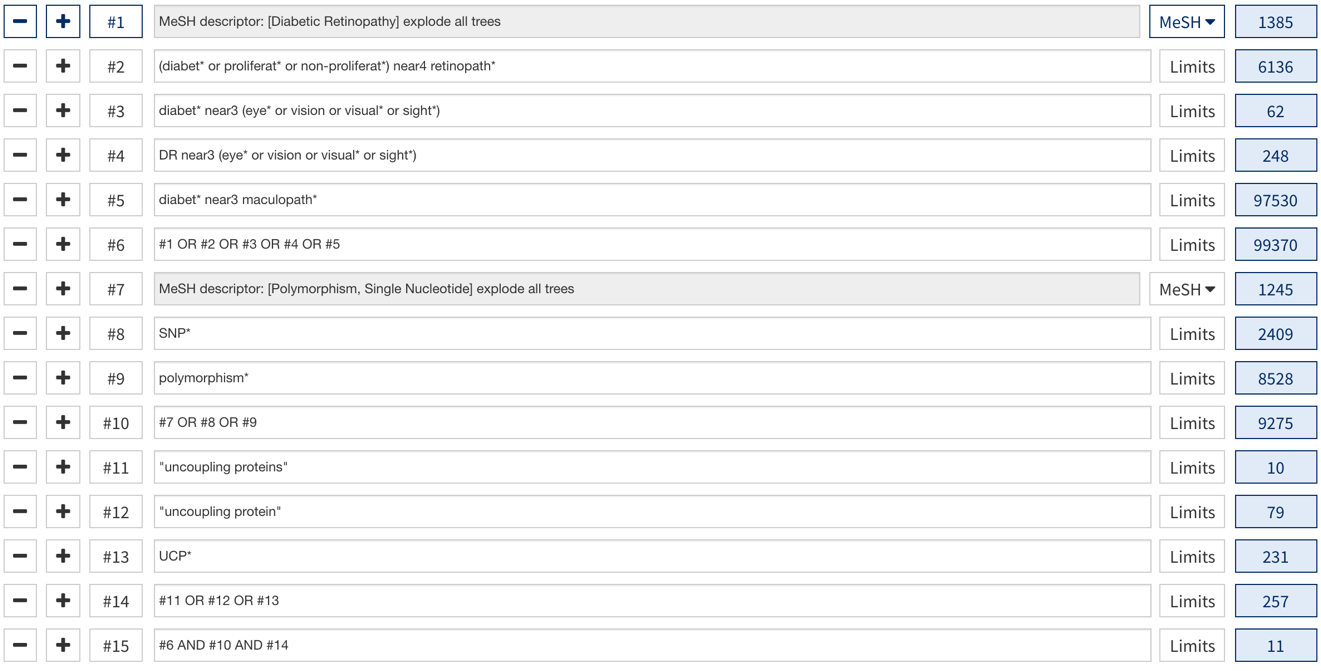

Supplement: Supplementary file 4 — Additional file 4: Table S1. Characteristics of other UCPs variants. Table S2. Analysis of the included studies by Newcastle-Ottawa quality assessment scale. Figure S1. Subgroup analysis of UCP1 rs1800592 by ethnicity. Figure S2. Subgroup analysis of UCP2 rs659366 by ethnicity. Figure S3. Subgroup analysis of UCP2 rs659366 by stage of DR. Figure S4. Sensitivity analyses of UCP1 rs1800592 in all genetic models. Figure S5. Sensitivity analyses of UCP2 rs659366 in all genetic models [file 12886_2021_1838_MOESM4_ESM.docx]
